# Methodological, planning and implementation challenges for rcts evaluating group interventions

**DOI:** 10.1186/1745-6215-14-S1-O124

**Published:** 2013-11-29

**Authors:** Daniel Hind, Chris Roberts, Kirsty Sprange, Rebecca Gossage-Worrall, Cindy Cooper, Stephen Walters

**Affiliations:** 1 Clinical Trials Research Unit, University of Sheffield, Sheffield, UK; 2 School of Community Based Medicine, University of Manchester, Manchester, UK; 3 School of Health and Related Research, University of Sheffield, Sheffield, UK

## 

Statistical concerns over the design and analysis of RCTs in which patients receive therapy as a group are well documented (see for instance, Roberts C, Clin Trials. 2005;2(2):152-62). By contrast, there is little discussion in the published literature of a range of other challenges to the success of such studies. These include how to plan for and manage: scheduling of participant recruitment, including matching the 'demand' of participants to the supply of interventionists where the RCT evaluates a new service; entry into the study of couples and twins; attrition from the study before and during the course, the threat to protocol compliance and group dynamics; the handling of 'en-masse' protocol violations; the collection of research data by therapists and the delivery of interventions by researchers. Group interventions are heterogeneous and there are a variety of ways in which these issues can be handled. For some, group administration of treatment may be just for convenience and efficiency; for others, the formation and composition of the group may be important for the function of an intervention in which interaction between participants is a key component.

This paper collates the experiences of registered CTU study managers and statisticians who have encountered and resolved these questions. Their UKCRCN portfolio studies include medical education, behaviour change and public health interventions delivered, variably, by NHS and University employees as well as third sector volunteers.

